# Dinner consumption and cardiovascular disease risk factors among a nationally representative sample of Iranian adolescents: the CASPIAN-III Study

**DOI:** 10.15171/jcvtr.2019.24

**Published:** 2019-06-30

**Authors:** Leila Azadbakht, Fahime Akbari, Mostafa Qorbani, Mohammad Esmaeil Motlagh, Gelayol Ardalan, Ramin Heshmat, Elnaz Daneshzad, Roya Kelishadi

**Affiliations:** ^1^Department of Community Nutrition, School of Nutritional Sciences and Dietetics, Tehran University of Medical Sciences, Tehran, Iran; ^2^Diabetes Research Center, Endocrinology and Metabolism Clinical Sciences Institute, Tehran University of Medical Sciences, Tehran, Iran; ^3^Department of Community Nutrition, School of Nutrition and Food Science, Isfahan University of Medical Sciences, Isfahan, Iran; ^4^Food Security Research Center, Isfahan University of Medical Sciences, Isfahan, Iran; ^5^Department of Public Health, Alborz University of Medical Sciences, Karaj, Iran; ^6^Department of Epidemiology, Non-Communicable Diseases Research Center, Endocrinology, and Metabolism Population Sciences Institute, Tehran University of Medical Sciences, Tehran, Iran; ^7^Department of Pediatrics, Faculty of Medicine, Ahvaz Jundishapur University of Medical Sciences, Ahvaz, Iran; ^8^Department of Pediatrics, Child Growth and Development Research Center, Research Institute for Primordial Prevention of Non-Communicable Disease, Isfahan University of Medical Sciences, Isfahan, Iran; ^9^Epidemiology Department, Chronic Diseases Research Center, Endocrinology& Metabolism Research Institute, Tehran University of Medical Sciences, Tehran, Iran

**Keywords:** Dinner, CVD Risk Factors, Adolescents

## Abstract

***Introduction:*** This cross-sectional study aimed to assess the association between cardiovascular disease (CVD) risk factors and dinner consumption in a nationally representative sample of Iranian adolescents.

***Methods:*** The present study was conducted on 5642 adolescents aged 10-18 years old in 27 provinces in Iran. The subjects were included applying by multistage random cluster sampling. Participants who ate ≥5 dinners during a week were considered as a dinner consumer.

***Results:*** Among 5642 subjects, 1412 (25%) did not consume dinner. Dinner consumers were less likely to be overweight or obese (*P * < 0.001) and abdominally obese (*P * < 0.001) as well as to have an abnormal level of HDL-C (*P * = 0.02). Dinner skipper youths had a higher risk for overweight or obesity (odds ratio [OR]: 1.62; 95% CI: 1.39-1.89) and abdominal obesity (OR: 1.59; 95% CI: 1.36-1.85) which remained significant after adjusting confounding factors (*P* <0001). No relationship was observed between dinner consumption and the rest of the CVD risk factors, neither in crude nor in adjusted models. A higher proportion of dinner-consumer adolescents had no CVD risk factors in comparison to dinner-skipper subjects (31.1% vs. 28%).

***Conclusion:*** Eating dinner might be inversely associated with some CVD risk factors among Iranian adolescents. Further prospective studies will need to prove this theory.

## Introduction

Cardiovascular diseases (CVDs) have remained high on a list of leading causes of death for more than a decade throughout the world.^1^ Although CVD mortality rate and CVD risk factors are decreasing in high-income countries, it tends to increase in low and middle-income countries.^2-5^ Additionally, reports indicated the growing trend for CVD prevalence in future; for example, it is estimated that 40.5% of the US population will have some form of CVD by 2030.^6^ Middle East countries have highest cardiovascular death rates through the world which possibly Iran has a higher burden in this region.^7^ Moreover, reports revealed a dramatically increasing rate for CVD risk factors in Iranian adolescents.^8^ Based on the results of the cohort studies, CVD risks in childhood and adolescence are predictors of CVD in adulthood.^9-12^

The CVD-diet relationship is assessed in several epidemiological surveys.^2^ Dietary factors affect CVD events^13^ and CVD risk factors.^14^ According to reports diet play an important role in developing or restricting CVD risk factors in adults^13,15-17^ as well as children and adolescents.^18-20^ Interventions to reduce CVD risk factors in regard to diet as a modifiable behavior have achieved favorable results.^21-23^ Earlier studies investigated the effect of a specific nutrient or component of diet like fat, carbohydrate, different fatty acids and salt on CVD risk factors.^13,24,25^ Whereas recent papers have mostly focused on dietary patterns^26^ and habits^27,28^ in relation to CVD risk factors. Eating frequency and main meals consumption have been linked to CVD and CVD risk factors.^28-30^ A recent paper showed an additional eating occasion was associated with lower levels of CVD risk factors including waist circumference, fasting glucose, fasting insulin, triglycerides (TG), TC, low-density lipoprotein cholesterol (LDL-C).^31^ An earlier survey on 10 years old children which aimed to investigate dietary variables in relation to CVD risk factors reported that most of the dietary cholesterol was received from breakfast and dinner.^32^ Another study among adults showed that dinner had the highest density for fat and cholesterol.^13^ In spite of being one of the main meals, dinner consumption is often neglected and few studies examined dinner consumption in relation to other variables. With regard to CVD risk factors, studies examined the just obesity-dinner relationship. Eating family dinner was negatively associated with being overweight among children and adolescents.^33-35^ However, one of the studies failed to show this association in longitudinal analysis among adolescents.^35^ Furthermore, eating family dinner was linked to better dietary intakes and habits^33,36^ and lower eating disorders.^37^

We are aware of no study evaluating the association between dinner consumption and CVD risk factors among adolescents. According to findings of different studies, Iranian children and adolescents prone to have abnormal CVD risk factors such as high prevalence of overweight and obesity,^38^ metabolic syndrome,^39^ and abnormal lipid profile.^40^ Therefore, we conducted this study to investigate whether there is any correlation between dinner consumption and each of CVD risk factors among a nationally representative sample of Iranian adolescents.

## Materials and Methods

### Subjects

The present study was performed using data of the “national survey of school student high-risk behaviors” (2009–2010) as the third survey of the school-based surveillance system titled CASPIAN-III *(“Childhood and Adolescence Surveillance and Prevention of Adult Non-communicable disease Study”)*. In spite of the published methodology in detail,^41^ a brief explanation is given therein. However, the sample size was calculated based on different variables but we considered the maximum sample size. Using multistage random cluster sampling 5642 Iranian school students aged 10 to 18 years were selected from elementary, intermediate, and high school schools among rural and urban areas of 27 provinces in Iran (with estimated 80% response rate, 20% was added to the sample size). Students with CVD or those on taking medicine were not enrolled in the present study due to exclusion criteria. Protocols of this study were approved by the ethics committees and other relevant national regulatory organizations. After explaining study objectives to students and parents, written informed consent and oral assent were obtained from parents and students, respectively.

### Assessment of anthropometric measurements

Weight was measured by the digital scale in barefoot and lightly dressed condition. Standing height of no-shoes-wearing subjects was marked on a stadiometer. Weight and height were measured twice, average to the nearest 0.2 kg and 0.2 cm, respectively. Body mass index (BMI) was calculated as the ratio of body weight in kilogram to height squared in meters. Waist circumference (WC) was measured using a nonelastic tape to the nearest 0.5 cm midway between the lower rib margin and iliac crest. In standing position widest hip was measured to the nearest 0.5 cm. According to the World Health Organization (WHO) guidelines for 10-19 years old adolescents, *“underweight, overweight and obesity were defined as BMI <5th, =85th-95th, and >95th percentiles, respectively”*.^42^ Abdominal obesity was predicated on WC (cm) divided by height (cm) >0.5.^43^

### Assessment of lipid profiles and blood pressure

To collect blood samples, participants were referred to the nearest health center to the school. Blood samples were collected from all participants. The samples of venous blood were taken after 12 h fast to assess lipid profiles including TC, LDL-C, HDL-C, TG and fasting plasma glucose (FPG). All biochemical analyses were performed in the Central Provincial Laboratory according to the standards of the National Reference Laboratory, a WHO collaborating center in Tehran. “*The borderline and risky cut-off points for this population were defined according to latest recommendation of the American Academy of Pediatrics as followed TC = 170-199 and ≥200 mg/dL, LDL-C = 110-129 and ≥130 mg/dL, HDL-C = 40-45 and <40 mg/dL, TG = 90-129 and ≥130 mg/dL, and FPG = 100-125 and > 125 mg/dL, respectively”.*^44^ Systolic blood pressure (SBP) and diastolic blood pressure (DBP) were measured twice with a suitable size of cuff for each student after a 5 minutes rest and in the sitting position. SBP was determined as the first sound which is clear and DBP as the disappearance of sound. The average of two-time measurements was considered for analysis.

### Assessment of dinner consumption and sociodemographic variables

The questionnaire applied in this study was designed based on the WHO STEPwise approach to Surveillance (STEPS) approved to non-communicable disease (NCD) (Tools ver 9.5) and WHO Global School Health Survey (GSHS). Some questions in regard to sociodemographic characteristics such as child’s birth weight and family dietary habits were included in the “parents’ questionnaire”.^45^ The validity and reliability of questionnaires have been confirmed in the first survey of this surveillance system.^45^ Smoking habits (yes or no), sleeping (hour/day), watching television (TV) (hour/day), and working with a computer (hour/day) were self-reported. To assess dinner-CVD risk factors, a binary variable applied through counting subjects that had ≥ 5 dinners during a week as a dinner consumer and < 5 as a dinner skipper.

### Assessment of Socioeconomic status

The method and variables which were used for calculating SES was approved in the previous international study for Iran.^46^ SES of family was constructed using some variables including parental occupation, parental education, possessing private car, school type (public/private), type of home (private/rented) and having personal computer at home. These variables were summarized in one main component named SES score using principal component analysis. A lower score corresponds to a lower SES.

### Statistical analysis

All analyses were done based on dinner consumption (yes/no) using SPSS for Windows software (version 16.0. SPSS, Chicago, IL). Descriptive variables such as age, weight and lipid profiles expressed as mean ± standard deviation (SD) and significant differences between means of 2 groups (dinner consumer or skipper) were examined employing Independent t test. Categorical variables like gender, normal BMI were represented as percentages and between-groups differences assessed by chi-square tests. Furthermore, the distribution of CVD risk factors according to dinner consumption was reported using chi-square tests. To evaluate the association between dinner consumption and CVD risk factors, logistic regression was employed with consideration of dinner consumer as reference and odds ratio (OR) with 95% confidence interval (CI) were reported. Adjusted model for sex, age, SES, physical activity, and smoking ^47^ was defined. Finally, to observe the overall effect of dinner on CVD risk factors chi-square tests were used.

**Figure 1:**
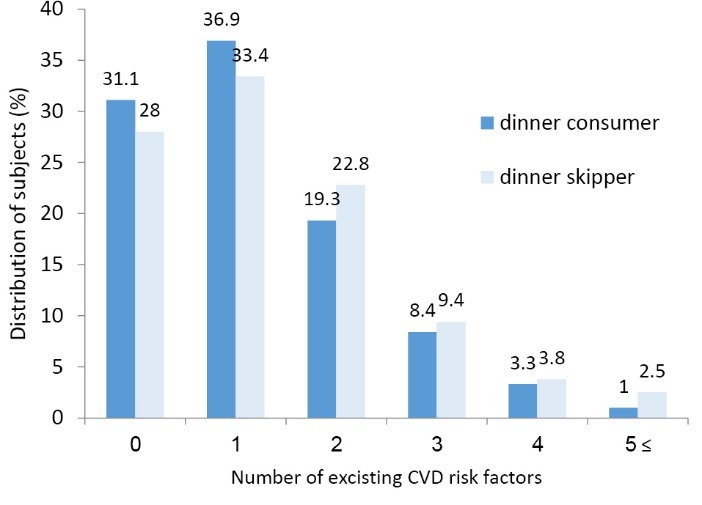
Distribution of adolescents regarding a number of identified CVD risk factors simultaneously according to dinner consumption; *P* = 0.007; eight CDV risk factors defined as underweight or overweight or obesity, abdominal obesity, high blood pressure, hypercholesterolemia, high LDL- cholesterol, Low HDL-cholesterol, Hypertriglyceridemia, High fasting plasma glucose.

## Results

A total of 2816 girls and 2826 boys aged 10 to 18 years old were examined in the present study. Among these population 4230 adolescents were dinner consumers while 1412 of individuals did not consume dinner; i.e. 25% of the population was not dinner consumers. Mean ± SD of general characteristics and CVD risk factors as well as the distribution of some features according to dinner consumption is shown in Table 1. Dinner consumers spent more hours sleeping, watching television (TV) and doing physical activity and fewer hours working with the computer (*P* < 0.001).

**Table 1:** General characteristics of the participants according to dinner consumption

**Variable**	**Dinner consumer**		***P***
	**Yes** ^ 1 ^ ** (n = 4230)**	**No (n = 1412)**	
Age (year)	14.85 ± 2 .42	14.37 ± 2.36	<0.001
Girls (%)	51.1	46.2	0.001
Urban (%)	70.2	67.3	0.040
Level of education (%) Primary school Secondary High school	30.4 34.4 35.2	41.1 31.2 27.6	<0.001
Weight (kg)	47.26 ± 14.93	46.80 ± 15.36	0.320
Height (cm)	154.88 ± 14.06	152.06 ± 13.48	<0.001
BMI (kg/m^2^)	19.30 ± 3.97	19.79 ± 4.42	<0.001
Normal BMI^3^ (%)	66.6	62.6	<0.001
Waist girth (cm)	68.81 ± 22.85	68.52 ± 12.20	0.652
Waist to Height ratio	0.44 ± 0.14	0.45 ± 0.07	0.151
Birth weight (kg) (%) <2500 g 2500-4000 g >4000 g	14.2 76.2 9.6	14.8 72.5 12.8	0.003
Systolic blood pressure (mm Hg)	103.42 ± 13.70	102.45 ± 14.29	0.033
Diastolic blood pressure (mm Hg)	65.97 ± 10.89	65.52 ± 10.72	0.191
Sleeping (hour/day)	9.12 ± 2.15	8.64 ± 2.44	<0.001
Watching TV (hour/day)	3.58 ± 1.21	3.38 ± 1.09	<0.001
Working with computer (hour/day)	1.97 ± 1.20	2.21 ± 1.18	<0.001
Physical activity (hour/day)	2.02 ± 1.21	1.60 ± 1.17	<0.001
Smoker (%)	12.7	12.4	0.851
Total cholesterol (mg/dl)	148.84 ± 32.23	147.36 ± 30.42	0.162
LDL-C (mg/dl)	83.98 ± 26.78	84.95 ± 28.98	0.391
HDL-C (mg/dl)	46.55 ± 14.47	45.24 ± 13.70	0.011
Triglycerides (mg/dl)	92.49 ± 41.49	94.41 ± 45.91	0.210
Fasting plasma glucose (mg/dl)	87.84 ± 13.68	86.94 ± 14.17	0.060
SGOT (U/L)	25.55 ± 13.49	26.35 ± 13.37	0.101
SGPT (U/L)	18.12 ± 11.27	18.32 ± 12.16	0.630

BMI: body mass index; LDL-C: low-density lipoprotein- cholesterol; HDL-C: high-density lipoprotein- cholesterol; SGOT: Aspartate aminotransferase; SGPT: Alanine Aminotransferase. ^1^Subjects were considered as dinner consumer if they had dinner ≥ 5 days a week. ^2^Values are mean ± SD, otherwise, it is indicated. ^3^Normal BMI: according to WHO criteria, 85th >BMI>5th.

Distribution of subjects with regard to CVD risk factors based on dinner consumption is given in Table 2. Dinner consumers were significantly more likely to be underweight (*P* = 0.004) and less likely to be overweight or obese (*P* < 0.001) and abdominally obese (*P* < 0.001) as well as to have an abnormal level of HDL-C (*P* = 0.02) compared with dinner skipper adolescents.

**Table 2:** Cardiovascular disease risk factors among dinner consumers and skippers

Variable	**Dinner consumer**				**P**
	**Yes (n = 4230)**		**No (n = 1412)**		
	**n**	**%**	**n**	**%**	
Underweight^1^ (%)	768	18.3	209	15.0	0.004
Overweight or obese^2^ (%)	634	15.1	312	22.4	<0.001
Abdominal obese^3^ (%)	594	14.1	289	20.7	<0.001
Hypertension^4^ Pre-HTN HTN	762 234	18.8 5.8	247 75	18.3 5.6	0.891
Total cholesterol^5^ (mg/dL) Borderline High	570 209	16.4 6.0	210 62	17.2 5.1	0.420
LDL-C^6^ (mg/dL) Borderline High	259 133	10.6 5.5	104 58	12.2 6.8	0.131
HDL-C^7^ (mg/dL) Borderline Low	477 1014	16.1 34.3	186 384	18.1 37.4	0.021
Triglycerides^8^ (mg/dL) Borderline High	988 474	28.7 13.8	344 183	29.6 15.7	0.151
Fasting plasma glucose^9^ (mg/dL) Borderline High	506 20	15.0 0.6	155 6	13.9 0.5	0.650

HTN: hypertension; LDL-C: Low-density lipoprotein–cholesterol; HDL-C: high-density lipoprotein-cholesterol Cut-offs for biochemical variables: Total Cholesterol; borderline= 170-199 and high ≥200 mg/dL, LDL-C; borderline= 110-129 and high ≥130 mg/dL, HDL-C; borderline= 40-45 and high <40 mg/dL, TG; borderline= 90-129 and high ≥130 mg/dL, and FPG; borderline= 100-125 and high > 125 mg/dL, ^1^Defines as BMI<5^th^ according to WHO criteria. ^2^Defines as according to WHO criteria, 95^th^ >BMI>85^th.^ ^3^Abdominal obese defined according to waist to height ratio >0.5. ^4^Pre-HTN: If BP confirmed 120/80-130/85, HTN: If BP confirmed >130/85. ^5^Total cholesterol: Borderline=170-199 mg/dL and high≥200 mg/dL. ^6^LDL- C: Borderline=110-129 mg/dL and high≥130 mg/dL. ^7^HDL- C: Borderline=40-45 mg/dL and low<40 mg/dL. ^8^Triglycerides: Borderline=90-129 mg/dL and high≥130 mg/dL. ^9^FPG; borderline: 100-125 mg/dL, high> 125 mg/dL.

Crude and multivariable-adjusted ORs for CVD risk factors according to dinner consumption are represented in Table 3. Dinner skippers were 21% less likely to be underweight than dinner-consumer participants (OR 0.79; 95% CI: 0.67-0.93). However, this relationship disappeared after adjusting for age, sex, physical activity, and smoking (OR 0.86; 95% CI: 0.55-1.12). Dinner skipper youths had a higher risk for overweight or obesity (OR 1.62; 95% CI: 1.39-1.89) and abdominal obesity (OR: 1.59; 95% CI: 1.36-1.85) which remained significant after adjusting (*P* < 0001). Additionally, dinner skipping was associated with 23 and 21% increased risk for borderline (OR: 1.23; 95% CI: 1.02-1.52) and low (OR: 1.21; 95% CI: 1.04-1.42) levels of HDL-C, respectively, in crude model among adolescents. After adjustments, the relationship disappeared for the low level of HDL-C, but for borderline levels, it remained strongly significant with 43% increased risk (OR: 1.43; 95% CI: 1.11-1.92; *P* = 0.006).

**Table 3:** Odds ratio (95% CIs) for having CVD risk factors among dinner consumers and skippers

**Variable**	**Dinner consumer**		***P***
	**Yes**	**No**	
**Underweight (%)** Crude Adjusted^1^	1.00 1.00	0.79 (0.67-0.93) 0.87 (0.66-1.14)	0.005 0.331
**Overweight or obese (%)** Crude Adjusted	1.00 1.00	1.62 (1.39-1.89) 1.53 (1.22-1.94)	<0.001 <0.001
**Abdominal obese (%)** Crude Adjusted	1.00 1.00	1.59 (1.36-1.85) 1.65 (1.30-2.10)	<0.001 <0.001
**Prehypertension** Crude Adjusted **HTN** Crude Adjusted	1.00 1.00 1.00 1.00	1.03 (0.88-1.21) 0.85 (0.67-1.10) 1.04 (0.80-1.37) 0.87 (0.60-1.28)	0.691 0.209 0.753 0.481
**Cholesterol (mg/dL)** Borderline Crude Adjusted High Crude Adjusted	1.00 1.00 1.00 1.00	0.95 (0.80-1.13) 1.19 (0.89-1.58) 1.18 (0.88-1.58) 1.45 (0.90-2.34)	0.550 0.221 0.271 0.120
**LDL-C (mg/dL)** Borderline Crude Adjusted High Crude Adjusted	1.00 1.00 1.00 1.00	0.84 (0.66-1.07) 0.98 (0.66-1.45) 0.77 (0.56-1.06) 1.67 (0.86-3.23)	0.150 0.920 0.111 0.120
**HDL-C (mg/dL)** Borderline Crude Adjusted Low Crude Adjusted	1.00 1.00 1.00 1.00	1.23 (1.02-1.52) 1.43 (1.11-1.92) 1.21 (1.04-1.42) 0.99 (0.77-1.26)	0.031 0.006 0.021 0.821
**TG (mg/dL)** borderline Crude Adjusted High Crude Adjusted	1.00 1.00 1.00 1.00	0.92 (0.79-1.07) 1.02 (0.81-1.27) 0.83 (0.67-1.01) 0.92 (0.69-1.22)	0.291 0.889 0.060 0.580
**FPG (mg/dL)** Borderline Crude Adjusted High Crude Adjusted	1.00 1.00 1.00 1.00	1.09 (0.90-1.33) 0.96 (0.72-1.26) 1.12 (0.45-2.79) 1.10 (0.36-3.32)	0.360 0.746 0.811 0.860

HTN: hypertension; LDL-C: Low-density lipoprotein–cholesterol; HDL-C: high-density lipoprotein-cholesterol Cut-offs for biochemical variables: Total Cholesterol; borderline= 170-199 and high ≥200 mg/dL, LDL-C; borderline= 110-129 and high ≥130 mg/dL, HDL-C; borderline= 40-45 and high <40 mg/dL, TG; borderline= 90-129 and high ≥130 mg/dL, and FPG; borderline= 100-125 and high > 125 mg/dL, ^1^ Adjusted for sex, age, socioeconomic status, physical activity, and smoking.

Distribution of adolescents regarding overall CVD risk factors based on dinner consumption is shown in Figure 1. As it is obvious, a higher proportion of the population in dinner consumer subjects has none of the risk factors or just 1 risk factors compared with dinner skippers, whereas having 2 or more risk factors simultaneously are more prevalent in dinner skipper than consumer individuals (*P* = 0.007).

## Discussion

In the present study which was performed among a nationally representative sample of 5642 Iranian adolescents, dinner skipping was prevalent and an inverse association was found between dinner consumption and some CVD risk factors including overweight or obesity, abdominal obesity, and HDL-C. Moreover, the results regarding overall CVD risk factors revealed that higher proportion of dinner-consumer adolescents had no or 1 CVD risk factors and a smaller proportion had 2 or more CVD risk factors simultaneously in comparison to dinner-skipper subjects.

To the best of our knowledge, regardless of a few studies examined the dinner-overweight relationship among adolescents,^33-35^ this study is the first paper trying to investigate the relationship between dinner consumption and CVD risk factors. Therefore, there is little opportunity to compare the findings of this study with similar ones and so inevitably, we review papers on breakfast or meal consumption despite the fact that consuming dinner may have entirely different outcomes.

Lower BMI and a higher proportion of normal BMI reported among subjects who consumed dinner could be attributed to dinner eating due to age-independent nature of BMI. Moreover, an inverse association between BMI and dinner consumption were revealed in this study is consistent with previous studies which were conducted in developing countries.^33-35^ In a study conducted among Japanese women, individuals who were late dinner consumer or consumed bedtime snakes were more likely to be overweight or obese.^48^ However, these women tend to skip breakfast and probably they were prone to be obese. Additionally, a recent report with a substantial sample size showed five-meal-a-day pattern strongly was associated with reduced risk of overweight and obesity in boys and girls.^49^ The direct association between skipping breakfast and increased risk of overweight and obesity is well-established especially in children and adolescents as a recently published systematic review and meta-analysis papers confirmed previous findings.^50,51^

On the other hand, the results of the present study suggested a novel inverse association between dinner consumption and abdominal obesity as well as low level of HDL-C. One important mechanism is related to energy balance according to the relationship between energy intake and energy expenditure. The previous study has indicated that regular meals consumption could increase energy expenditure and contribute to reducing weight.^52^ The inverse association between consuming 5 meals per day and abdominal obesity were reported only in boys.^49^ Also, a study in 13 303 twenty years old individuals revealed that people who eat dinner alone are more likely to be obese than individuals who eat dinner with family.^53^ However, reduced risk for abdominal obesity and abnormal level of HDL-C might attribute to the other healthy lifestyles. It should be considered that although dinner consumer subjects reported more hours of physical activity and fewer hours working with a computer, they spent more hours sleeping and watching TV. In addition, OR for mentioned risk factors after adjusting potential confounders like physical activity remained significant.

In spite of a higher risk of being underweight in dinner eater, it cannot be deduced that dinner consumption is associated with underweight as the association is disappeared after adjusting some confounders. Previous studies mostly focused on overweight as CVD risk factors and the underweight-meal consumption relationship has not been fully examined. A recent study among 1-7 years old children suggested skipping breakfast was associated with underweight.^54^

This study failed to reach to a significant association between dinner consumption and other CVD risk factors i.e. hypertension, TC, LDL-C, TG, and FPG levels. Likewise, a population-based survey could not find any association between eating 5 meals daily and other risk factors after adjustment for potential confounders except for overweight and abdominal obesity.^49^ A recent data revealed a negative association between frequency of eating breakfast and blood glucose, TG and HDL-C levels in obese children and adolescents even after adjusting some confounders.^55^ In contrast, another study which was conducted in adults reported that eating breakfast regularly was associated with evaluated serum TG.^56^

Considering the number of CVD risk factors identified in adolescents according to dinner eating led to interesting results. In the present study, a higher percentage of dinner eater subjects had no or 1 risk factors and fewer suffered from 2 or more risk factors compared to adolescents’ skipped dinner.

Although, we considered the frequency of eating dinner, quantity, and quality of dinner also play an important role in developing CVD risk factors as suggested in the recent papers (in 2013). One study among overweight women diagnosed as having metabolic syndrome which compared 2 weight loss isocaloric diet, i.e. high caloric intake in breakfast (700 kcal breakfast, 500 kcal lunch, 200 kcal dinner) versus dinner (200 kcal breakfast, 500 kcal lunch, 700 kcal dinner) for 12 weeks revealed that participants who received high-calorie breakfast had a greater amount of weight loss and reduced waist circumference and lower levels for fasting glucose and triglyceride in comparison to high-calorie dinner consumers.^57^ Examining the effects of eating carbohydrates and proteins mostly at lunch or dinner among overweight/obese men suggested that eating carbohydrates mostly at lunch and protein mostly at dinner had an unfavorable impact on glucose homeostasis.^58^

Besides the short-term effects of meal consuming on weight loss, a recent report demonstrated that regular meal frequency could even modify the effect of common genetic variants on BMI in adolescents.^59^ Previous studies proved that more frequent meals pattern has favorable effects on CVD risk factors which attributed to reduced concentrations of serum insulin.^60,61^ Therefore, we assumed that eating dinner regularly has similar results through the same mechanism.

Some limitations should be considered regarding this study. The first one may be noted is the cross-sectional nature of this study which could not lead to inferring a causal relationship. Due to incomplete data on some dietary factors, adjusted models for dietary variables could not be used to extract the full effects of dinner frequency on CVD risk factors. Converting dinner frequency to a binary variable might leads to probable misclassification. Maybe using an ordinal variable and comparing the highest versus lowest might result in more significant associations.

A representative sample of the Iranian population and large sample size as well as controlling some potential confounders could be addressed as strength points for this study.

In conclusion, eating dinner is inversely associated with some CVD risk factors among Iranian adolescents. Therefore, more emphasis should be given to dinner consumption of adolescents. However, because of limited investigations on this issue, further studies with the prospective design are needed to confirm our finding.

## Ethical approval

The study was approved by Research and Ethics Committee of Isfahan University of Medical Sciences; oral assent and written informed consent were obtained from students and their parents, respectively.

## Competing interests

All authors declare no competing financial interests exist.

## Funding

This study was conducted as part of a national surveillance program, supported by the Ministry of Health.

## Acknowledgements

The authors are thankful for all individuals which participated in this study.
